# Limit, lean or listen? A typology of low-value care that gives direction in de-implementation

**DOI:** 10.1093/intqhc/mzy100

**Published:** 2018-05-07

**Authors:** Eva W Verkerk, Marit A C Tanke, Rudolf B Kool, Simone A van Dulmen, Gert P Westert

**Affiliations:** Radboud University Medical Center, Radboud Institute for Health Sciences, IQ Healthcare, Nijmegen, The Netherlands

**Keywords:** low-value care, overuse, de-implementation, disinvestment, de-adoption

## Abstract

**Background:**

Overuse of unnecessary care is widespread around the world. This so-called low-value care provides no benefit for the patient, wastes resources and can cause harm. The concept of low-value care is broad and there are different reasons for care to be of low-value. Hence, different strategies might be necessary to reduce it and awareness of this may help in designing a de-implementation strategy. Based on a literature scan and discussions with experts, we identified three types of low-value care.

**Results:**

The type ineffective care is proven ineffective, such as antibiotics for a viral infection. Inefficient care is in essence effective, but is of low-value through inefficient provision or inappropriate intensity, such as chronic benzodiazepine use. Unwanted care is in essence appropriate for the clinical condition it targets, but is low-value since it does not fit the patients’ preferences, such as a treatment aimed to cure a patient that prefers palliative care. In this paper, we argue that these three types differ in their most promising strategy for de-implementation and that our typology gives direction in choosing whether to limit, lean or listen.

**Conclusion:**

We developed a typology that provides insight in the different reasons for care to be of low-value. We believe that this typology is helpful in designing a tailor-made strategy for reducing low-value care.

## Introduction

Overuse of unnecessary care is widespread around the world and especially prevalent in high-income countries [1, 2]. Experts estimate that ~10–30% of all healthcare practices have little or no benefit to the patient [3, 4]. Apart from wasting limited resources, these so-called low-value care practices may cause physical, psychological and financial harm to patients [1]. For example, an unnecessary CT-scan exposes the body to harmful radiation and overuse of antibiotics contributes to antibiotic resistance at population level. Berwick and Hackbarth estimated that between $107 billion and $389 billion was wasted on low-value care in the USA in 2011 [5]. Reducing low-value care is therefore a step towards the triple aim in healthcare: improving the experience of care and the health of populations, and reducing its costs [6]. Hence, there is an increasing number of initiatives around the world to identify and reduce low-value care [1, 7], the largest of them being Choosing Wisely [8].

The concept of low-value care is broad and listed low-value services vary, ranging from routine transthoracic echocardiograms [9] to the chronic use of benzodiazepines [10] and curative treatment for patients that prefer palliative care [11]. These cases of low-value care have different contexts and different reasons for being of low-value, enable different perspectives by diverse stakeholders and require different strategies for de-implementation. Just as in implementation [4], one size does not fit all in de-implementation and tailoring your strategy to the context of the low-value care practice is important. We are convinced that being aware of the reason for care to be of low-value is important in selecting a strategy.

To the best of our knowledge, there is no literature that reports taking this into account in developing a strategy for reducing low-value care. The aim of this paper is to introduce a typology of low-value care that creates awareness of the wide range of low-value care and provides direction in how to reduce it.

## What is low-value care?

What low-value care entails depends on the definition of value. Literature shows different definitions for low-value care that contain several elements [12–18]; low-value care is care: that provides minimal or no health benefit; which benefit does not weigh up to the harms; which benefit does not weigh up to the costs; that is less cost-effective than alternative care, and that does not fit the preferences of the patient. There is no definition that encompasses all elements. Therefore, we will use the following definition of low-value care: ‘care that is unlikely to benefit the patient given the harms, cost, available alternatives, or preferences of the patient’. This definition includes care that is low-value from both the patients’ and societal perspective.

Low-value care is also being addressed in other terms, such as overuse, which is often mentioned next to underuse (failing to provide care when it would have produced a favorable outcome) and misuse (selecting high-value care but not delivering to its full potential due to preventable complications) [12]. The related terms overtreatment and overtesting indicate the inappropriate delivery of particular types of services [1]. Another related term, overdiagnosis, occurs when people without symptoms are diagnosed with a disease that ultimately will not cause symptoms or early death [19].

In this paper, we focus on care that is proven to be of low-value and of which the physician can predict it is of low-value at the time of deciding to deliver the specific care practice. We do not focus on care that has unknown effectiveness and care that appeared to be of no value after it had been used. However, determining if a care practice is unlikely to benefit the patient on beforehand can be hard. Often there is a lack of sound scientific evidence, for example, because studies lack an appropriate comparator or relevant and long-term outcome measures [20]. Drugs and medical devices can be authorized for the market based on this weak evidence. And even when there is sufficient evidence, using it to predict for an individual patient whether a practice is of low-value or not could also be hard.

## Current typologies

**Figure 1:**
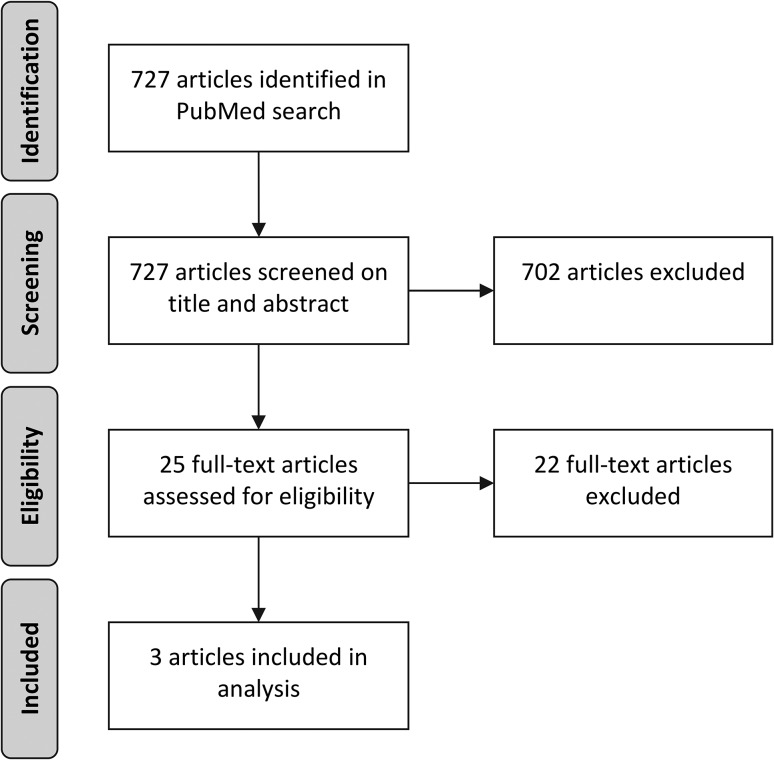
Flowchart literature scan.

We reviewed scientific literature on low-value care of the past 10 years and found three papers that describe a typology or framework with different types of low-value care related to the reason for being low-value [13, 21, 22]. We searched PubMed on 28 March 2017 with the following search strategy and included articles from 01 January 2007: (low-value care[tiab] OR lower-value care[tiab] OR unnecessary care[tiab] OR overuse[tiab] OR overdiagnosis[tiab] OR Medical Overuse[Mesh]) AND (framework[tiab] OR types[tiab] OR typology[tiab] OR classification[tiab]). E.W.V. and S.A.vD. screened all articles independently and discussed for final inclusion. See Fig. 1 for a flowchart of this process. We included articles that describe different types of low-value care related to the reason for being low-value. We excluded papers without typologies and papers with typologies that did not provide insight into the reason for being low-value, such as type of care (diagnostics, treatment or prevention), costs and effects of care, and barriers and facilitators for reducing low-value care. Wennberg identified three types of unwarranted variations in care; effective care, preference-sensitive care and supply-sensitive care [23]. However, these unwarranted variations include both overuse of low-value care and underuse of high-value care, while we focus on care that is proven of low-value.

The found typologies describe several reasons for care to be of low-value, such as when care ‘occurs too frequently’ [13], ‘is not clinically indicated for the patient’s symptom or diagnosis’ [13], ‘is delivered in the wrong doses or duration’ [21], ‘has a cheaper, equally effective alternative’ [21] or ‘has a close benefit-risk balance in mild cases’ [22].

The typologies all include categories focused on the value of a service from a medical perspective. However, none of the typologies include the option of care being low-value due to the patients’ preferences. Since patient preferences are recognized in the definitions of low-value care and evidence based practice, and are recognized by Choosing wisely as being an important component of avoiding overuse [24], the current typologies do not represent the full spectrum of low-value care. In addition, two typologies include categories that do not match our definition of low-value care [21, 22], We would categorize ‘Not receiving a medicine that is clinically needed’ as underuse, and ‘canceled procedures’ and ‘potentially cosmetic interventions’ are not necessarily low-value according to our definition. Some categories within the typologies have the same underlying cause for being low-value. For example, the categories ‘services that are not matched to the patient’s risk of disease’ and ‘when the patient has contraindications that increase the risk of the service’ both represent care whose benefits do not outweigh the risks. Lastly, the typologies do not facilitate the selection of a promising strategy for reducing low-value care. Each typology offers insight in low-value care, but they do not comprise the full spectrum of low-value care and they do not give direction to reducing low-value care. Therefore, we developed a new typology.

## Introduction of a new typology

**Figure 2:**
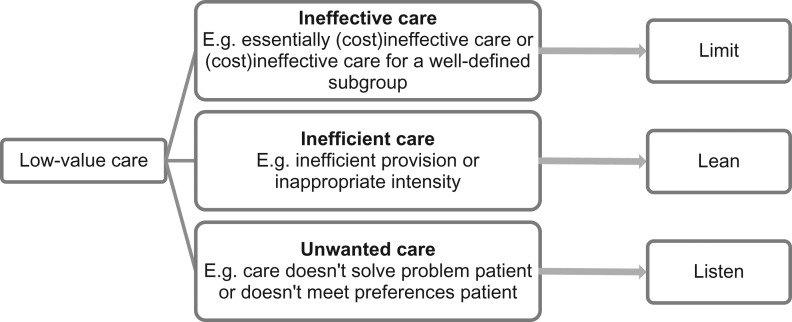
Typology of low-value care.

Based on our definition of low-value care and in collaboration with five clinicians and researchers with expertize on low-value care or implementation, we created three types of low-value care related to their reason for being of low-value. Figure 2 shows our typology. The category ineffective care is of low-value from a medical perspective. It includes care that is proven (cost)ineffective for a certain condition or which benefit does not weigh up to the harms according to scientific standards, for the majority of the population or a well-defined subgroup. Examples are shaving before an operation, the use of antibiotics in children with upper respiratory tract infections and routine echocardiography for asymptomatic patients.

The category inefficient care is of low-value from a societal perspective. It includes care that is in essence effective for the targeted condition, but becomes of low-value through inefficient provision or inappropriate high intensity or duration. Examples of inefficient provision are duplication of diagnostic tests and removing stitches in hospital instead of general practice. Examples of inappropriate intensity are routine use of ‘last-resort’ antibiotics, chronic benzodiazepine use and prolonged catheterization.

The category unwanted care, lastly, is of low-value from the patients’ perspective. Like ‘inefficient care’ it is in essence effective for the targeted condition, but becomes low-value because it does not solve the individual patients’ problem or does not fit the individual patient’s preferences. Examples are vaccines and blood transfusions for patients with certain religious beliefs, chemotherapy for a patient that prefers palliative care, or surgery while the patient prefers conservative treatment. This category is probably the least well-known and least well-studied type of low-value care, because it can only be identified and measured by assessing the patient’s values.

An example to illustrate this typology is the use of an magnetic resonance imaging (MRI) scan in a patient with a lumbal hernia. An MRI scan may have been low-value because the scan was not indicated (ineffective); because the scan had been done before (inefficient) or because the outcome of the scan would not alter treatment anyway: the patient prefers conservative treatment over an operation (unwanted). Logically, the strategy to reduce unnecessary MRI scans in each of the three options differs.

## Using the typology in reducing low-value care

We argue that these three types differ in their most promising strategy for de-implementation. For the category ‘Ineffective care’, it can be clearly determined which patients do and do not need to receive certain care. This enables macro-level strategies enacted by the government or national institutes with consequences for the whole community, such as market withdrawal or exclusion from the benefit package, which make care inaccessible or unprofitable. These are strong incentives and can be a successful and sustainable addition to a de-implementation process. However, policy changes could be difficult to achieve. Other strategies for reducing ineffective care are incorporation of do-not-do recommendations in clinical practice guidelines and protocols or installing barriers or alerts in electronic patient records when a low-value care practice is ordered. For example, a study installed soft- and hard-stop computer alerts when metformin was ordered inappropriately [25]. The key word for de-implementing this kind of low-value care is ‘limit’.

‘Inefficient care’ is caused by inefficient organization and lack of cooperation. Market withdrawal or exclusion from the benefit package are not possible, since this care is in essence effective and still needs to be delivered. A promising and sustainable strategy here lies in hospitals or regional networks reorganizing care and facilitating communication between healthcare providers. Duplication of imaging for example might be solved by better information transmission between electronic patient files. Another example is a study that reduced the high intensity of routine laboratory tests by implementing a new ordering system in which each test needs to be ordered individually instead of in groups [26]. The key word for de-implementing this kind of low-value care is ‘lean’.

Since ‘unwanted care’ depends on the preferences and values of the patient, limiting or reorganizing care for all patients is not appropriate. A promising strategy for reducing unwanted care is facilitating shared-decision-making and sufficient communication between patient and caregiver. It is important that patients are well-informed before making a decision and empowered to be more involved in their healthcare, although this could be difficult because it requires time and skills from the caregiver. An example is a study that reduced unwanted prostate cancer screening by providing patients with a decision aid and educating physicians [27]. The key word for de-implementing this kind of low-value care is ‘listen’.

Incorporating the reason for care to be of low-value in developing de-implementation strategies is important but not sufficient. Other contextual factors (e.g. local organizational structure, culture, available time and money) play an important role and need to be taken into account in a full-grown strategy. This means that facilitators and barriers that either stimulate or impede wise choices need to be tackled [28, 29]. The driving factors can be different for every low-value care practice and can include fear of litigation, financial incentives, pressure from patients or lack of consultation time [30]. Also, combining multiple strategies is generally more effective than a single strategy [18]. Even when taking all these elements into account, achieving sustainable change is hard and takes determination, time and money. This is a challenge we need to face in order to reduce low-value care and improve healthcare.

## Conclusion

We have developed a typology with three types of low-value care related to their reason for being of low-value that describe the full spectrum of low-value care according to our definition. Care can be of low-value because it is ineffective, inefficient and unwanted. Recognition of these reasons may help to stimulate the debate on how to reduce low-value care. Since for different types of low-value care, different types of action may be the most promising target for sustainable de-implementation, this typology may help in developing a tailor-made strategy. Low-value care is an increasing problem in western countries and there is an urge to take action. Reducing low-value care increases the quality and safety of care and reduces costs, and should be on the agenda in every country on policy, organizational and professional level. In addition, countries should focus on preventing low-value care by investing in proper research and stricter market authorization. We are positive that this typology will give insight in low-value care and guide healthcare providers, policy makers and researchers in the challenge of de-implementing low-value care in many countries.
